# The Impact of Antibiotic Resistance in Childhood Campylobacter Infections Before and After the COVID-19 Pandemic in the Southeast Region of Romania

**DOI:** 10.3390/antibiotics14020170

**Published:** 2025-02-10

**Authors:** Cristina Chiurtu, Elena Mocanu, Bogdan Florentin Nitu, Ana Maria Iancu, Cristina Maria Mihai, Mara Andreea Cambrea, Raluca Mihai, Mihaela Mavrodin, Anca Daniela Pînzaru, Ramona Mihaela Stoicescu

**Affiliations:** 1 Departament of Infectious Diseases, Faculty of General Medicine, “Ovidius” University, 900470 Constanta, Romania; chiurtu.cristina@yahoo.ro (C.C.); amiancu1016@gmail.com (A.M.I.); raluca.mihai91@yahoo.com (R.M.); cln_mihaela@yahoo.com (M.M.); 2 Department of Public Health and Management, Faculty of General Medicine, “Ovidius” University, 900470 Constanta, Romania; elena.mocanu@univ-ovidius.ro; 3 Departament of Pediatrics, Faculty of General Medicine, “Ovidius” University, 900470 Constanta, Romania; cristina_mihai@365.univ-ovidius.ro; 4 Departament of Microbiology and Immunology, Faculty of Pharmacy, “Ovidius” University, 900470 Constanta, Romania; ramona.stoicescu@univ-ovidius.ro

**Keywords:** pandemic, antibiotics, Campylobacter, resistance, children

## Abstract

The world has changed forever as a result of the COVID-19 pandemic. Antimicrobial resistance is a primary global health concern that places a significant financial and health burden on nations. Patients with Campylobacter-caused infections were the subject of the retrospective investigation. The data show that children aged 1–6 are the most commonly affected by Campylobacter enteritis. Resistance levels fluctuated over the course of the two periods. Nine isolates were sensitive to macrolides, and only one was responsive to tetracycline, which indicated inadequate sensitivity across all classes throughout the pandemic. This pattern raises serious concerns about the potential impact on public health. Tetracyclines and fluoroquinolones rank highest in terms of bacterial resistance. Regardless of the species, macrolides remain a practical and sufficient treatment for Campylobacter enteritis. Reassurance is still provided by much lower numbers in the post-pandemic period. There is no evidence to support the alarming claims made in the international literature about macrolides in Romania.

## 1. Introduction

The COVID-19 epidemic has irrevocably altered the globe. Medical practice has been profoundly impacted, particularly in pediatrics. Due to the change in global antibiotic usage recommendations, a reassessment of recognized infections in modern frameworks has become necessary. Antimicrobial resistance is an important health issue that costs nations money and causes significant health impacts. The development of antimicrobial resistance is linked to increased antibiotic use. We highlight the consequences of antibiotic use, particularly with regard to increasingly common microorganisms like Campylobacter. It is an important issue that requires immediate attention and action.

11 May 2023 marks the end of the COVID-19 epidemic. It represents a significant point of human, medical, and scientific demarcation. Being understood as a point of rebirth, knowing the impact of antibiotic therapy in medical activity becomes an essential element of analysis [1].

Alignment with the standards of the European community entails the need to regulate the consumption of antibiotics. Romania is among the most affected countries, with over 3% of its population using antibiotics daily [2]. According to the most recent data from 2021, Romania is the EU member with the highest consumption of systemic antibacterials in the hospital and community sectors [3]. Infection with Campylobacter has become one of the most prevalent diseases in children, regardless of their financial situation [4,5,6,7]. *C. jejuni* is the most incriminated species in triggering episodes of gastroenteritis. There are documented cases of recurring or chronic infections with these bacteria, highlighting the need for immediate action [7].

In 2023, the number of cases of Campylobacter intestinal infection in Romania rose by 2.8 compared to the pandemic years (2020–2022). Greece, Poland, Bulgaria, and Romania had the lowest rates during the pre-pandemic years (2018–2019), whereas the Czech Republic and Luxembourg had the highest cases [8,9].

**Figure 1:**
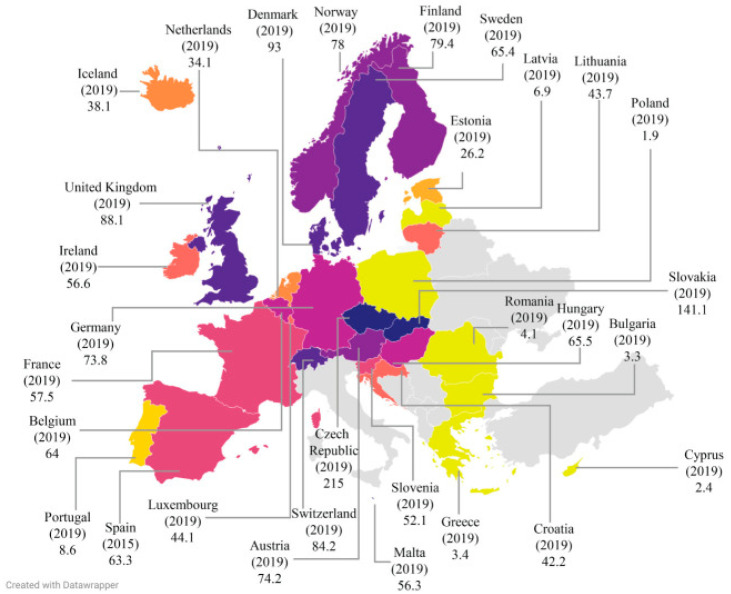
The incidence rates of campylobacteriosis in Europe and around the world are reported per 100,000 people, according to Liu [7].

Figure 1 Reported prevalence of campylobacteriosis worldwide.

Certain species of Campylobacter can travel corkscrew-like because they have flagellums on one or both ends of their bodies [7,10]. *Campylobacter jejuni* is the primary cause of human campylobacteriosis cases, typically manifesting as gastroenteritis. Clinical manifestations vary from diarrhea, fever, and abdominal pain to intractable vomiting and a permanent feeling of nausea. The illness may last for several weeks, with median infection lengths ranging from 7 to 31 days; however, a significant proportion of infections persists for more than a month [7,11,12,13]. While the health effects of acute campylobacteriosis are extensively established, the significance of persistent or recurring Campylobacter infection remains barely comprehended. International data show the detrimental effects of intestinal infections caused by Campylobacter. Due to inflammatory intestinal syndromes caused by this bacteria, developmental disorders occur in children living in resource-poor areas of the world, which also results in decreased weight gain and impaired linear growth [7,13].

## 2. Results

In adults, its prevalence is modest according to international data [14]. Thus, we decided to focus on studying the impact of campylobacter infection in children. According to the specific parameters, enterocolitis was identified in over 875 pediatric patients. Only 154 children satisfied the inclusion requirements after using the acceptability templates from the examined batch. The database encompasses one hundred and fifty-four patients diagnosed with Campylobacter-caused acute enterocolitis between 2018 and 2024. This substantial sample size was key to ensuring the findings’ validity and underscored the research’s importance.

One of the most important steps in comprehending the information supplied by the group being analyzed was the exploration of the sociodemographic data. These data are instrumental in understanding the transmission path and implementing new preventative strategies. The foundation of all efforts to curb the rise in fatalities from preventable diseases, primarily caused by bacterial resistance, lies in the collective responsibility to use antibiotics judiciously.

Gastroenteritis, caused by infections with Campylobacter species, typically presents as diarrhea that may or may not be bloody, stomach pain, and emesis. A Campylobacter infection can resemble intussusception, appendicitis, or inflammatory bowel disease in children. Furthermore, 82% had a temperature higher than 39.8 degrees Celsius and 18% had a fever below 39.0 degrees Celsius. Every patient had a fever prior to or at admission, with a median duration of three to five days. Moreover, 73.5% of the patients tested positive for stool occult blood, while 28.5% of the patients complained of bloody diarrhea at admission. According to the preliminary lab results, 28.5% and 61.1% of patients had elevated WBC counts > 20,000/mm^3^ and CRP levels ≥ 10 mg/dL. Vomiting and emesis were present in more than 59.86%. Diarrhea stools (more than 8/day) were the most common manifestation (95.8%).

According to the data, Campylobacter enteritis predominantly affects children aged 1–6, with an average age of 3.3 and a deviation of 2.08 years. The group that met the study’s acceptability conditions varied between 1 and 16 years. Given the median age of two, it is likely that half of the patients were younger than two, highlighting the population’s diversity.(Table 1)

The difference in Campylobacter enteritis prevalence between urban and rural areas has been closing since 2020, marking a significant shift in the disease’s prevalence. Romania belongs to the group of nations with low to moderate levels of economic development. The number of patients admitted to the study falls into the rural area category due to limited accessibility to healthcare services and inadequate adherence to food and personal hygiene regulations. Bacterial enteritis has been prevalent in urban areas. The rural environment has always stood out with a higher number of enterocolitis cases, including cases of Campylobacter gastroenteritis. This pattern could also be observed in the pre- and post-COVID-19 periods.

The distribution was balanced by gender, with 51.3% of the population being girls (*n* = 79) and 48.7% being boys (*n* = 75). Regarding origin, 49% of the patients were from rural areas and 51% were from metropolitan areas. Crucially, there were no appreciable differences across the groups, boosting confidence in the validity of the results. The distribution of gender and environment of origin was approximately equal.

Of the 154 patients examined, 127 (82.5%) needed to be admitted to the hospital, while 27 (17.5%) received outpatient care. The length of hospitalization for those who needed it ranged from 1 to 11 days, with an average of 4.4 days and a median of 4 days. The standard deviation, which was 1.8 days, shows a moderate degree of variability across instances.

Out of every case that was taken into consideration, only 27 (17.53%) indicated a potential path of transmission. This underscores the potential for further research in this area. Moreover, 35% reported a recent travel history and 55.8% reported consuming water from unknown sources.

Three days is the median (interquartile range) amount of time that passed between the start of symptoms and the first visit to the doctor, although it may be longer than 5 days.

The database includes 154 patients with acute enterocolitis brought on by Campylobacter between 2018 and 2024. The distribution of cases by year showed a significant increase in the first years of the study (2018: 38 cases; 2019: 56 cases). The aggressiveness of cases of Campylobacter infections is proven by the recrudescence of cases in the immediate post-pandemic period (2023: 35 cases; 2024: 25 cases).

To observe the trend of these infections, the cases were divided into two analysis intervals, all directly dependent on the period of the COVID-19 pandemic. Division I concerned the pre-pandemic period (2018–2019, *n* = 94; 61%), while the second division represented the post-pandemic period (between 2023 and 2024, *n* = 60; 38.9%). This temporal division aims to build a clear picture of the years of analysis with particular socio-demographic and financial implications. The pandemic period was strictly focused on the prevention, early diagnosis, and treatment of infection with the coronavirus, with the rest of the pathologies remaining in the shadows.

The analysis of the distribution of Campylobacter species (*C. coli n* = 17; *C. jejuni n* = 46; *C.* spp. *n* = 91) highlighted significant differences between the pre-pandemic and post-pandemic periods (Table 2).

**Figure 2:**
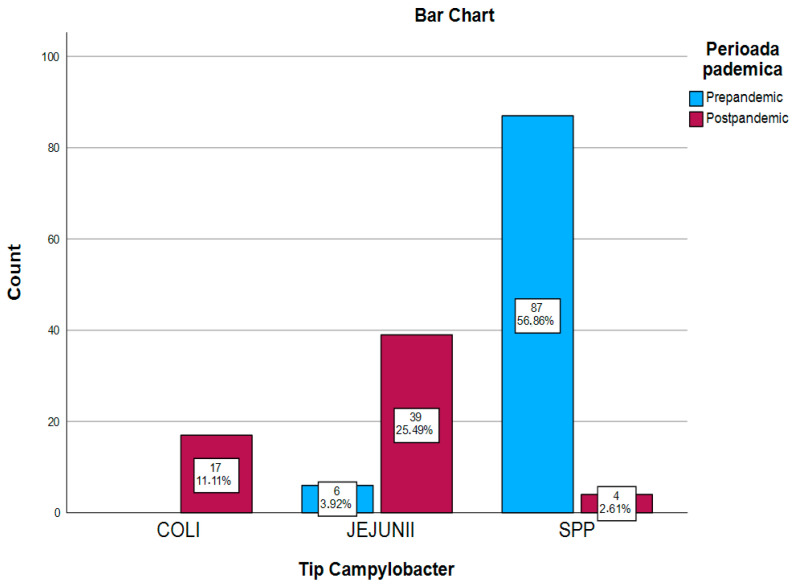
Distribution of Campylobacter species according to the pandemic period.

In the pre-pandemic period (2018–2019), *Campylobacter* spp. was responsible for 92.5% of cases (*n* = 87), while *Campylobacter jejuni* was present in a small number (*n* = 7, 7.4%). *Campylobacter coli* was not detected during this period (Figure 2).

In the post-pandemic period (2023–2024), species distribution changed significantly. *Campylobacter jejuni* increased in 41.4% of cases (*n* = 39) and *Campylobacter coli* showed a significant increase, representing 28.3% of cases (*n* = 17) (Figure 2).

A statistically significant variation in species distribution between the two periods was confirmed by the Chi-square tests (x^2^ = 11,514, *p* < 0.001), suggesting important changes over time. Specifically, *Campylobacter jejuni* exhibited a higher prevalence in the post-pandemic period compared to the pre-pandemic period. *Campylobacter* spp. showed elevated levels during the pre-pandemic period in contrast to the post-pandemic interval. These findings highlight notable shifts in species distribution across the two analyzed time frames (Table 3).

During the trial, macrolides showed effectiveness against campylobacteriosis, and no resistant bacteria were found. Moreover, 152 (98.7%) of the 154 isolates, which are individual bacterial cells or colonies that have been separated from a sample, were found to be responsive. Since all of the isolates in this class of antibiotics were sensitive and showed no fluctuation, the Chi-square test was applicable; the value indicated no statistically significant link.

During the 2018–2019 pre-pandemic phase, all 87 isolates exhibited sensitivity. The sensitivity showed no signs of fluctuation over the post-pandemic years (2023–2024), providing a stable and consistent set of results.

There were notable differences in tetracycline resistance between the periods analyzed. The Chi-square test, a statistical method used to determine whether there is a significant association between two variables, correlated the resistance and study periods (χ^2^ = 0.28, *p* = 0.596). Of the 138 isolates tested, 43 (31.2%) were susceptible and 95 (68.8%) were resistant. Minor variations were observed in the association between analyzed periods and sensitivity to tetracyclines; however, these were not statistically significant (*p* = 0.596).

Fluctuating levels of resistance were recorded during the two periods. In the pre-pandemic period (2018–2019), a significant percentage of isolates were resistant (58), and 29 were susceptible. In the post-pandemic period (2023–2024), resistance decreased slightly, with 37 resistant isolates and 14 susceptible isolates.

The Chi-square test (Cramer’s V = 0.045) revealed a very weak correlation between periods and tetracycline resistance. This finding suggests that factors outside the analyzed periods, such as the use of antibiotics in human or veterinary medicine, may significantly influence the evolution of resistance, requiring further investigation.

During the research, only 10.3% of the isolates were susceptible to fluoroquinolones, indicating a high incidence of resistance (89.7%). What is more concerning is that this resistance persisted across different periods, and the statistical analysis showed no significant differences. In the pre-pandemic period (2018–2019), 10.3% of isolates were sensitive (*n* = 9) and 89.7% (*n* = 78) were resistant. In the post-pandemic period (2023–2024), resistance remained at a similar level, with 89.7% (*n* = 52) of isolates being resistant and only 10.3% (*n* = 6) being sensitive. (Table 4)

The Kruskal–Wallis test (H = 3.63, *p* = 0.604 for tetracyclines; H = 6.07, *p* = 0.299 for fluoroquinolones) did not indicate a significant variation in the prevalence and antibiotic sensitivity of Campylobacter species between pre-pandemic and post-pandemic periods. The statistical evidence presented previously only strengthens antibiotic therapy’s effectiveness as an elective treatment for this kind of illness. The pre-pandemic period exhibited the highest sensitivity, with a preponderance of macrolide sensitivity (87/87 sensitive strains), followed by the post-pandemic phase (65/65 sensitive strains). All classes had poor sensitivity after the pandemic, with only six strains responsive to fluoroquinolones and fourteen strains sensitive to tetracyclines.

*Campylobacter coli*, which showed complete susceptibility to macrolides (16/16 strains), provides a reassuring indication of the effectiveness of this antibiotic. This species also had a higher prevalence (28.3%) in the post-pandemic era (2023–2024).

Predominant in the post-pandemic era, *Campylobacter jejuni* exhibited full sensitivity to macrolides (39/39 strains) but also showed a concerning high resistance to tetracyclines (26/30 strains), highlighting the urgent need for alternative treatments.

The pre-pandemic prevalence of *Campylobacter* spp. was 94.3% (*n* = 87); in the post-pandemic era, the prevalence significantly dropped to 6.7% (*n* = 8), indicating a potential positive impact of the pandemic on infectious diseases. The bacteria also exhibited high sensitivity to macrolides in both periods (87/87 strains pre-pandemic and 6/6 strains post-pandemic).

Susceptibility data for fluoroquinolones were not available for *Campylobacter* spp. or *Campylobacter coli*. However, a distinct trend was observed for *Campylobacter jejuni*, where the post-pandemic period showed a higher proportion of fluoroquinolone-sensitive cases compared to the pre-pandemic period. These findings may reflect shifts in antibiotic usage patterns or evolving resistance mechanisms influenced by pandemic-related factors (Table 5).

Fluoroquinolone resistance was significantly higher in *Campylobacter* spp. during the pre-pandemic period (76/80 cases) compared to the post-pandemic period (4/80 cases). This marked decrease in resistance in the post-pandemic era could reflect shifts in antimicrobial usage policies, changes in healthcare practices, or other factors influenced by pandemic-related interventions (Table 6).

These findings demonstrate how bacterial sensitivity varies significantly for other antibiotics, particularly tetracyclines, yet remains consistently high for macrolides throughout time.

## 3. Materials and Methods

This study, conducted retrospectively at the Constanta Infectious Diseases Hospital, Romania between January 2018 and August 2024, focused on patients diagnosed with infections caused by Campylobacter bacteria. The study concentrated on identifying Campylobacter-caused enterocolitis and the impact of antibiotic treatment on resistance levels. These diagnoses were confirmed using the gold standard of microbiological cultures from stool samples. The analysis was based on a batch of 154 samples of fecal stool samples matter examined between 2018 and 2024. Following the parents’ or caregivers’ signed approval, a pre-structured questionnaire was used to gather sociodemographic, environmental, behavioral, and clinical data with the assistance of qualified doctors.

Testing for antimicrobial susceptibility: Strains of Campylobacter were identified on agar plates. In accordance with the European Committee for Antimicrobials’ protocol, the Kirby–Bauer disc diffusion method was used to test for antimicrobial susceptibility to tetracycline, fluoroquinolones (ciprofloxacin), and macrolides (erythromycin and azithromycin).

In accordance with the EUCAST guidelines, the diffusometric technique was used to screen the isolates of *Campylobacter jejuni* and *Campylobacter coli* for antibiotic sensitivity. The inhibitory zone was measured in millimeters (mm), and antibiograms were conducted for ciprofloxacin, macrolides, and tetracycline. The results were interpreted based on the particular standards for every antibiotic and species: Erythromycin: *C. jejuni*: sensitivity ≥ 20 mm, resistance < 20 mm, *C. coli*: sensitivity ≥ 24 mm, resistance < 24 mm, Tetracycline: sensitivity ≥ 24 mm, resistance < 30 mm, Ciprofloxacin: sensitivity: diameter > 50 mm, resistance < 26 mm.

### Analysis of Statistics

Microsoft Excel created a database that helped consolidate the gathered data. Demographic information, sex, time frame, and Campylobacter type (*Coli*, *Jejuni*, Spp.) were all included. SPSS, a potent tool for data processing and statistical analysis, was used to conduct statistical analyses.

## 4. Discussion

This study examined campylobacteriosis laboratory monitoring data from January 2019 to October 2024 in Romania. The results provide insight into several important facets of this illness, such as multifactorial resistance patterns, sources, demographic trends, and notification rates. Due to the significant impact on public health and the potential for treatment ineffectiveness, the problem of antibiotic resistance in Campylobacter species is quickly growing in importance. The most effective treatments for Campylobacter infections, tetracyclines, and macrolide antibiotics are now dealing with rising macrolide resistance, which could have major consequences if ignored, which highlights the urgent need for coordinated efforts to reduce the emergence and spread of resistant strains [15,16,17].

Until the pandemic, the number of gastroenteritis cases stayed constant in Romania. However, the number sharply decreased after restricting measures were imposed. Examining the COVID-19 phenomenon alongside intestinal pathology highlights the sharp decline in instances [14,18,19]. To put it another way, practices like hand cleanliness and social distancing can lessen both direct transmission between people and transmission across contaminated surfaces. This will impact how the gastroenteritis-causing agent spreads. Halichidis et al. and Cambrea et al. also stressed the role that foodborne pathogens play in the spread of the bacteria that cause gastroenteritis episodes. The information is also applicable to the ongoing research [20,21].

Typically, a Campylobacter infection leads to a mild, self-limiting enteritis that necessitates supportive care. The early application of treatment measures such as probiotics, oral or intravenous rehydration, and antimotility medications are key elements in reducing the disease episode [21,22,23,24].

A foodborne bacterium, campylobacter, is mainly found in raw poultry products [13,24]. Furthermore, 75.3% of chicken consumption cases were attributed to cooking at home, 23.5% to take-out food, and 16.1% to restaurant eating. These findings suggest that the percentage of delivery consumption was higher. The imposition of isolation measures has determined a vertiginous increase in the consumption of food already prepared through take-out services. The imposition of isolation rules led to a drastic decrease in the number of gastroenteritis cases and poor compliance with hygiene rules. In other words, foods or ingredients with hazy hygienic standards or unclear expiration dates may be used as more individuals order food for delivery. Additionally, because of the nature of food delivery, customers cannot directly inspect the company’s cooking methods and hygiene status [14,25,26,27].

Gastroenteritis, the most prevalent disease caused by *C.* spp., *jejuni*, and *C. coli*, presents with severe symptoms in children, including fever, vomiting, diarrhea, and abdominal pain. The study emphasizes how urgent this problem is. At least half of the afflicted youngsters have bloody stools, and the illness can cause severe dehydration [28]. Infection with Campylobacter has been associated with serious complications such as encephalopathy, seizures, and meningismus, often accompanied by high fevers. These findings, extrapolated from the international literature, underscore the severity of the situation [29,30]. Bacteremia, although rare, is a serious concern, particularly in immunocompromised individuals [30].

Wi et al. reported clinical manifestations that align with those observed in the present study [31]. Fever and hematochezia are prominent features of the clinical presentation. Shane et al. highlighted the importance of comprehensive bacterial screening in diagnosing enteritis cases, particularly those characterized by bloody stools. This screening should encompass Salmonella, Yersinia, Shigella, Clostridium difficile, Escherichia coli, especially Shiga toxin (STEC), and Campylobacter. The predominant clinical manifestations of these bacteria include fever, bloody stools, and dehydration [32].

Additionally, a Campylobacter infection can resemble other gastrointestinal disorders. Infants with only bloody stools and vomiting without fever may have intussusception mistaken for Campylobacter enteritis. In older children, acute Campylobacter ileocolitis might resemble appendicitis by causing severe lower-right-quadrant discomfort without diarrhea [33]. The infection often progresses from the small bowel distally, while patients with severe Campylobacter enteritis may have colitis and bloody diarrhea, which can be confused with inflammatory bowel disease [22,34]. To rule out other intra-abdominal processes, like intussusception, imaging can be useful. Histologic analysis should make it simpler to differentiate between the chronic inflammatory changes and the acute inflammatory changes of a Campylobacter infection, which are marked by neutrophil infiltration and mucosal destruction. Furthermore, it has been suggested that a Campylobacter infection may play a role in the onset of inflammatory bowel disease [22,35].

Fever and gastrointestinal symptoms need cautious anamnesis. As was already established, these symptoms can conceal surgical diseases, particularly in females. Cambrea et al. highlighted the diverse etiology of fever in Romania. For instance, Mediterranean spotted fever has an endemic evolution in our territory, and its primary symptoms in youngsters can be gastrointestinal [36].

According to the research, preschoolers between the ages of one and six are the age group most affected by Campylobacter enteritis. This research emphasizes the necessity for the global health community to take a cooperative approach. Research throughout the world, including from Australia, supports these findings, demonstrating that children under five are the most impacted group [37]. Other research has shown a bimodal age distribution, with children under 5 and individuals between 15 and 45 being the most affected [14,37,38]. Furthermore, while the overall frequency of bacterial enteritis is decreasing, the prevalence of Campylobacter enteritis is on the rise across all age groups. This underscores the importance of disease management and proper hygiene, particularly in children under five, who are experiencing the highest rate of increase. The changing disease epidemiology is believed to be the driving force behind this trend [14,38,39].

These reports reveal a striking gender disparity in Campylobacter enteritis, with girls being more susceptible than boys. These findings challenge the established data published by Green et al. and Cho et al., sparking a new avenue of research and discussion [14,40,41].

The prevalence of Campylobacter enteritis in rural areas, as the data suggest, is higher than in urban areas. This complexity in the demographic data contradicts the findings of other international research, which indicate a higher prevalence in urban environments. Understanding the disease is further intellectually stimulated by the interaction of variables like climate and availability of healthcare [42,43,44,45].

According to many other surveys [46], hospital visits for mild illnesses have dramatically declined since COVID-19. Nonetheless, both the average length of stay and the number of bacterial enteritis patients have been increasing. These events are linked to reports of increasing antibiotic-resistant bacterial enteritis [47]. Every year, the average length of time for Campylobacter enteritis increases. The change in focus to other pathologies, once the pandemic was over, led to an increase in cases of bacterial resistance to antibiotics and an increase in the hospitalization period. It leads to increased costs in terms of hospitalization and treatment [48]. All of these factors constitute an alarm signal in the application of new prevention and treatment strategies. As seen in this study, the pediatric population has a more extended period of hospitalization, mainly girls and adolescents.

The analysis of Campylobacter strains in parallel with the effectiveness of antibiotic therapy highlights increased effectiveness among macrolides but with progressively increasing resistance in the case of fluoroquinolones and tetracyclines. The increase in resistance to antibiotics of strains of *Campylobacter* spp. determines the most urgent problem [8]. Antibiotic resistance in *Campylobacter* spp. is a growing problem that urgently requires concerted efforts. The data obtained in this study highlight that, after analyzing the temporal distribution of cases from the point of view of antibiotic resistance, there was a higher resistance to fluoroquinolones and tetracyclines in the pre-pandemic period—the most resistant strain was *Campylobacter* spp. The recent increase in ciprofloxacin resistance, a key antibiotic for treating Campylobacter infections, underscores the severity of the issue [15,16]. According to international research, resistance to aminoglycosides remains low [49]. Therefore, tracking the degree of resistance in circulating isolates is a critical aspect of campylobacteriosis surveillance. Urgent attention is required for the significant levels of priority antibiotic resistance, particularly in *C. coli*, found in Portuguese Campylobacter isolates with multiple drug resistance patterns [17,50].

As can be seen from the statistical analysis of Campylobacter spp, in the post-pandemic period, it no longer shows increased resistance, and the number of cases of multi-drug resistance drops precipitously. However, when we observe the trend of *Campylobacter coli* and *Campylobacter jejuni*, a migration of the pattern is noted. Thus, these two strains show a marked increase in antibiotic resistance post-pandemic. While *C. jejuni* and *C. species* showed resistance to fluoroquinolones and tetracycline alone, *C. coli*’s status is comparable to that of *C. jejuni*. *C. coli* had a significant degree of sensitivity to macrolide but a very high level of resistance to both tetracycline and fluoroquinolones. Multiple drug resistance was frequently seen in isolates of *C.* spp. However, it was rare in *C. jejuni*. The high level of resistance in *C.* spp. is a grave concern. Major signs of concern are the situations of combined antibiotic resistance to fluoroquinolones and macrolides [51].

Throughout the study, none of the analyzed species showed resistance to macrolides. European reports in the field of animals indicate an increase in cases of *Campylobacter jejuni* and *Campylobacter coli* infections with multi-drug resistance (at least two antibiotics) [43].

The CDC states that tetracycline, fluoroquinolones (ciprofloxacin), and macrolides (erythromycin) are the recommended antimicrobial treatments for Campylobacter species [52,53]. All of the isolated Campylobacter species in this study (100%) were erythromycin-sensitive, comparable to other studies from Europe and Africa but different from other studies with different resistance patterns [54]. As a counterpoint, in studies developed in Iran, Hawassa (55%) and Bahirdar (17.6%) showed resistance to macrolides [55,56]. Abay et al. found that the Campylobacter species had a resistance of 35.7% to ciprofloxacin, which is less than the 61.7% seen in Iranian investigations [57,58]. Additionally, the tetracycline resistance rate was 21.4%, roughly equivalent to a study performed in Iran (20.5%) [56,57] but less than in research from Poland (39.1%) [59], Jimma (39.5%) [60], Gondar (56.8%) [61], and Bahirdar (22.2%) [62] and 49.18% stronger resistance than the Ugandan study [63]. The European Food Safety Authority (EFSA) demonstrated in 2021 that ciprofloxacin and erythromycin resistance rates in *C. jejuni* isolated from human samples differed significantly among European nations. Germany displayed more variances, with resistance to ciprofloxacin ranging from 1.95 to 92.2% and erythromycin ranging from 2.2% to 66.6%. In Slovenia, the resistance rate was notably high at 14.2% for erythromycin and 86.9% for ciprofloxacin. Similarly, resistance rates in Romania ranged from 0.1% to a concerning 74.7% [64].

International studies draw attention to the significant increase in Campylobacter infection cases associated with increased antibiotic resistance. To confirm the veracity of the data obtained, we collected reports similar to those obtained in this study (resistance to fluoroquinolones between 24% and 65%) from international publications on different continents (UK, South Korea, Israel, and Peru) [65,66,67,68,69].

On the other hand, China (87%) and Japan (90%) bring to the fore the significant increase in the rate of resistance to ciprofloxacin [69,70,71]. The American Antibiotic Resistance Monitoring System (NARMS) has been emphasizing since 2015 an increase in resistance to ciprofloxacin in terms of *C. jejuni* (25.3%) and *C. coli* (39.8%) strains. In this study, macrolides showed 100% efficacy on all analyzed strains. A maximum of 10% was reported in 2015 for *Campylobacter jejuni* strains in neighboring regions [66,67]. As a particularity, the resistance to macrolides of *C. jejuni* has the lowest resistance rate in the entire southeast region [65,68]. International studies, even those outside Europe, report a low incidence of macrolide resistance, as seen in South Korea (0.8%), the UK (2.2%), Thailand (12.5%), China (21.8%), and India (22.2%) [67,68,69,70,71,72].

By making a statistical comparison with a study from 2012 developed by Cambrea et al., we can say that in the Constanta region, resistance to antibiotics in cases of gastroenteritis in children maintains a linear trend, with resistance to tetracyclines being noted from that moment [73].

The use of antibiotics to promote growth in poultry production has been linked to the increase in fluoroquinolone and macrolide resistance [69,74,75,76]. However, since there are no explicit laws governing the amount of antibiotics animals can receive in Romania, this is merely an assumption rather than a verifiable fact. We can only extrapolate data from other nations, which strictly regulate these dosages and overlay the incidents reported by the youngsters. What is becoming true is that poultry breeding is still practiced in households, and poultry can be sold without a veterinary check in advance.

## 5. Conclusions

This pattern raises significant warning signs. Resistance to antibiotics, predominantly fluoroquinolones and tetracyclines, was demonstrated in the present work. Much lower values in the post-pandemic period still provide reassurance. Although the international literature raises alarm signals regarding macrolides in Romania, no data support this idea.

## Figures and Tables

**Table 1:** Distribution according to age, sex, and territorial belonging.

	2018	2019	2023	2024	*p* Value
**Age group**					
	Remittance	Remittance	Remittance	Remittance	-
1–6 years	3.21	2.15	1.78	1.89	<0.001
7–12 years	1.89	2.55	1. 42	1.24	
13–16 years	1.21	1.45	0.89	0.75	
**Sex distribution**					
	Remittance	Remittance	Remittance	Remittance	-
Boys	0.83	0.61	0.15	0.33	<0.001
Girls	0.81	0.85	0.24	0.35	
**Territorial distribution**					
	Remittance	Remittance	Remittance	Remittance	-
Urban	1.89	1.75	0.88	0.75	
Rural	2.36	2.22	0.98	0.92	

The territorial and gender distribution respects the data reviewed in 2020 applicable to Dobrogea—Constanta region. ANOVA test was performed when the number of categories exceeded two; ANOVA—Analysis of variance per 100,000 cases.

**Table 2:** Distribution of Campylobacter species according to the pandemic period.

Campylobacter Strains	Pre-Pandemic *n* = 94	Post-Pandemic *n* = 60	Total *n* = 154
***Campylobacter coli***	0 (0%)	17 (28.3%)	17 (11%)
***Campylobacter jejuni***	7 (7.4%)	39 (41.4%)	46 (30%)
***Campylobacter* spp.**	87 (92.5%)	4 (12%)	91 (59%)

**Table 3:** Chi-square test.

Chi-Square Tests			
	Value	df	Asymptotic Significance (2-Sided)
Pearson Chi-Square	115,142 ^a^	2	<0.001
Likelihood Ratio	136,770	2	<0.001
Linear-by-Linear Association	101,909	1	<0.001
*n* of Valid Cases	153		

^a^ 0 cells (0.0%) have expected counts of less than 5. The minimum expected count is 6.67.

**Table 4:** Distribution according to antibiotic usage before, during, and after pandemic.

Antibiotics	Period	Resistance (*n*)	Sensible (*n*)	Total (*n*)
Macrolide	Pre-pandemic	0	93	93
	Post-pandemic	0	59	59
Tetracyclines	Pre-pandemic	60	30	90
	Post-pandemic	35	13	48
Fluoroquinolones	Pre-pandemic	79	9	88
	Post-pandemic	51	6	57

**Table 5:** Resistance profile to antimicrobial agents of *Campylobacter* spp., *jejuni*, and *Campylobacter coli* strains.

	Sensitivity			*p*
	*Campylobacter* spp.	*Campylobacter jejuni*	*Campylobacter* *coli*	
**Macrolides**	Pre-pandemic: **87** Post-pandemic: **4**	Pre-pandemic: **6** Post-pandemic: **39**	Pre-pandemic: **0** Post-pandemic: **16**	0.002
Fluoroquinolones	Pre-pandemic: **8** Post-pandemic: **0**	Pre-pandemic: **1** Post-pandemic: **2**	Pre-pandemic: **0** Post-pandemic: **2**	<0.001
Tetracyclines	Pre-pandemic: **29** Post-pandemic: **1**	Pre-pandemic: **1** Post-pandemic: **8**	Pre-pandemic: **0** Post-pandemic: **3**	<0.001

**Table 6:** Chi-Square test for fluoroquinolone.

Chi-Square Tests				
Sensitivity to Fluoroquinolone		Value	df	Asymptotic Significance (2-Sided)
Sensitivity	Pearson Chi-Square	9870 ^b^	2	0.007
	Likelihood Ratio	12,229	2	0.002
	Linear-by-Linear Association	8865	1	0.003
	*n* of Valid Cases	13		
Intermediar	Pearson Chi-Square	.^c^		
	*n* of Valid Cases	2		
Rezistent	Pearson Chi-Square	102,497 ^d^	2	<0.001
	Likelihood Ratio	121,554	2	<0.001
	Linear-by-Linear Association	88,630	1	<0.001
	*n* of Valid Cases	130		
Total	Pearson Chi-Square	113,906 ^a^	2	<0.001
	Likelihood Ratio	135,783	2	<0.001
	Linear-by-Linear Association	99,195	1	<0.001
	*n* of Valid Cases	145		

^a^. Zero cells (0.0%) have expected counts less than 5. The minimum expected count is 6.68. ^b^. Five cells (83.3%) have expected counts less than 5. The minimum expected count is 0.62. ^c^. No statistics are computed because Tip Campylobacter and pandemic periods are constants. ^d^. Zero cells (0.0%) have expected counts less than 5. The minimum expected count is 5, 10.

## Data Availability

Ethics commission available from Hotararea nr50/4.12.2024.

## References

[1] End of the Federal COVID-19 Public Health Emergency (PHE) Declaration. https://archive.cdc.gov/www_cdc_gov/coronavirus/2019-ncov/your-health/end-of-phe.html.

[2] Ministry of Health Romania (2021). Official Note to Update the Government Decision Regarding Forming the National Committee on the Prevention and Limiting of Healthcare Associated Infections. https://oldsite.ms.ro/wp-content/uploads/2021/08/NOT%C4%82-DE-FUNDAMENTARE-CN-IAAM.docx.

[3] European Centre for Disease Prevention and Control (ECDC) (2022). Antimicrobial Consumption Dashboard (ESAC-Net). https://qap.ecdc.europa.eu/public/extensions/AMC2_Dashboard/AMC2_Dashboard.html#eu-consumption-tab.

[4] Schiaffino F., Colston J.M., Olortegui M.P., Yori P.P., Mourkas E., Pascoe B., Lima A.A., Mason C.J., Ahmed T., Kang G. (2024). The epidemiology and impact of persistent *Campylobacter* infections on childhood growth among children 0–24 months of age in resource-limited settings. eClinicalMedicine.

[5] Cody A.J., McCarthy N.M., Wimalarathna H.L., Colles F.M., Clark L., Bowler I.C.J.W., Maiden M.C.J., Dingle K.E. (2012). A longitudinal 6-year study of the molecular epidemiology of clinical *Campylobacter* isolates in Oxfordshire, United Kingdom. J. Clin. Microbiol..

[6] Dingle K.E., McCarthy N.D., Cody A.J., Peto T.E., Maiden M.C. (2008). Extended sequence typing of *Campylobacter* spp., United Kingdom. Emerg. Infect. Dis..

[7] Liu F., Lee S.A., Xue J., Riordan S.M., Zhang L. (2022). Global epidemiology of Campylobacteriosis and the impact of COVID-19. Front. Cell Infect. Microbiol..

[8] Baltoiu M., Gradisteanu Pircalabioru G., Cristea D., Sorokin M., Dragomirescu C.C., Stoica I. (2024). Genetic Diversity, Virulence, and Antibiotic Resistance Determinants of *Campylobacter jejuni* Isolates in Romania. Pathogens.

[9] European Centre for Disease Prevention and Control (2016). EU Protocol for Harmonised Monitoring of Antimicrobial Resistance in Human Salmonella and Campylobacter Isolates—June 2016.

[10] Lastovica A.J., On S.L., Zhang L. (2014). The family *Campylobacteraceae*. Prokary.

[11] Galanis E. (2007). *Campylobacter* and bacterial gastroenteritis. Can. Med. Assoc. J..

[12] Amour C., Gratz J., Mduma E., Svensen E., Rogawski E.T., McGrath M., Seidman J.C., McCormick B.J., Shrestha S., Samie A. (2016). Epidemiology and impact of *Campylobacter* infection in children in 8 low-resource settings: Results from the MAL-ED study. Clin. Infect. Dis..

[13] Haque A., Platts-Mills J.A., Mduma E., Bodhidatta L., Bessong P., Shakoor S., Kang G., Kosek M.N., Lima A.A.M., Shrestha S.K. (2019). Determinants of *Campylobacter* infection and association with growth and enteric inflammation in children under 2 years of age in low-resource settings. Sci. Rep..

[14] Cho H., Lee S.H., Lee J.H., Lee S.J., Park S.C. (2023). Epidemiologic and Clinical Features of *Campylobacter* Enteritis Before and During COVID-19 in Korea. J. Korean Med. Sci..

[15] Rivera-Mendoza D., Martínez-Flores I., Santamaría R.I., Lozano L., Bustamante V.H., Pérez-Morales D. (2020). Genomic Analysis Reveals the Genetic Determinants Associated with Antibiotic Resistance in the Zoonotic Pathogen *Campylobacter* spp. Distributed Globally. Front. Microbiol..

[16] Ge B., Wang F., Sjölund-Karlsson M., McDermott P.F. (2013). Antimicrobial Resistance in *Campylobacter*: Susceptibility Testing Methods and Resistance Trends. J. Microbiol. Methods.

[17] Duarte A., Pereira L., Lemos M.L., Pinto M., Rodrigues J.C., Matias R., Santos A., PTCampyNet, Oleastro M. (2024). Epidemiological Data and Antimicrobial Resistance of *Campylobacter* spp. in Portugal from 13 Years of Surveillance. Pathogens.

[18] Ahn S.Y., Park J.Y., Lim I.S., Chae S.A., Yun S.W., Lee N.M., Kim S.Y., Choi B.S., Yi D.Y. (2021). Changes in the occurrence of gastrointestinal infections after COVID-19 in Korea. J. Korean Med. Sci..

[19] Pines J.M., Zocchi M.S., Black B.S., Carlson J.N., Celedon P., Moghtaderi A., Venkat A. (2021). Characterizing pediatric emergency department visits during the COVID-19 pandemic. Am. J. Emerg. Med..

[20] Cambrea S.C., Petcu L.C., Mihai C.M., Hangan T.L., Iliescu D.M. (2019). Influence of Environmental Factors About evolution of Shygellosis in Constanta County of Romania. J. Environ. Prot. Ecol..

[21] Smith G.S., Blaser M.J. (1985). Fatalities associated with *Campylobacter jejuni* infections. JAMA.

[22] Same R.G., Tamma P.D. (2018). *Campylobacter* Infections in Children. Pediatr. Rev..

[23] Santos J., Musta V., Luca C.M., Belei O.A., Cambrea S.C. (2021). Randomized, placebo-controlled trial of xyloglucan and gelose for the treatment of acute diarrhea in children. Expert. Rev. Gastroenterol. Hepatol..

[24] Diaconu S., Cambrea S.C., Petcu L.C., Rugină S. (2017). Aspects of Nosocomial Gastroenteritis with Rotavirus in Children hospitalized in Constanta—Romania. Acta Medica Mediterr..

[25] Bolton D.J. (2015). *Campylobacter* virulence and survival factors. Food Microbiol..

[26] Rha J.Y., Lee B., Nam Y., Yoon J. (2021). COVID-19 and changes in Korean consumers’ dietary attitudes and behaviors. Nutr. Res. Pract..

[27] Unnikrishnan A., Figliozzi M.A. (2020). A Study of the Impact of COVID-19 on Home Delivery Purchases and Expenditures.

[28] Blaser M.J., Berkowitz I.D., LaForce F.M., Cravens J., Reller L.B., Wang W.L. (1979). *Campylobacter* enteritis: Clinical and epidemiologic features. Ann. Intern. Med..

[29] Jones P.H., Willis A.T., Robinson D.A., Skirrow M.B., Josephs D.S. (1981). *Campylobacter* enteritis associated with the consumption of free school milk. J. Hyg..

[30] Levy I., Weissman Y., Sivan Y., Ben-Ari J., Scheinfeld T. (1986). Acute encephalopathy associated with *Campylobacter* enteritis. Br. Med. J..

[31] Wi D., Choi S.-H. (2023). Antibiotic Prescribing Practices and Clinical Outcomes of Pediatric Patients with *Campylobacter* Enterocolitis. Children.

[32] Shane A.L., Mody R.K., Crump J.A., Tarr P.I., Steiner T.S., Kotloff K., Langley J.M., Wanke C., Warren C.A., Cheng A.C. (2017). 2017 Infectious Diseases Society of America Clinical Practice Guidelines for the Diagnosis and Management of Infectious Diarrhea. Clin. Infect. Dis..

[33] Puylaert J.B., Vermeijden R.J., van der Werf S.D., Doombos L., Koumans R.K. (1989). Incidence and sonographic diagnosis of bacterial ileocaecitis masquerading as appendicitis. Lancet.

[34] Loss R.W., Mangla J.C., Pereira M. (1980). *Campylobacter* colitis presenting as inflammatory bowel disease with segmental colonic ulcerations. Gastroenterology.

[35] Castaño-Rodríguez N., Kaakoush N.O., Lee W.S., Mitchell H.M. (2017). Dual role of *Helicobacter* and *Campylobacter* species in IBD: A systematic review and meta-analysis. Gut.

[36] Cambrea S.C., Badiu D., Ionescu C., Penciu R., Pazara L., Mihai C.M., Cambrea M.A., Mihai L. (2023). Boutonneuse Fever in Southeastern Romania. Microorganisms.

[37] Moffatt C. (2021). Examining the Epidemiology of Campylobacteriosis in Australia. Ph.D. Dissertation.

[38] Nelson W., Harris B. (2011). Campylobacteriosis rates show age-related static bimodal and seasonality trends. N. Z. Med. J..

[39] Chen J., Sun X.T., Zeng Z., Yu Y.Y. (2011). *Campylobacter* enteritis in adult patients with acute diarrhea from 2005 to 2009 in Beijing, China. Chin. Med. J..

[40] Prescott S.L., Wegienka G., Logan A.C., Katz D.L. (2018). Dysbiotic drift and biopsychosocial medicine: How the microbiome links personal, public and planetary health. Biopsychosoc. Med..

[41] Green M.S., Schwartz N., Peer V. (2020). Sex differences in Campylobacteriosis incidence rates at different ages—A seven country, multi-year, meta-analysis. A potential mechanism for the infection. BMC Infect. Dis..

[42] Lévesque S., Fournier E., Carrier N., Frost E., Arbeit R.D., Michaud S. (2013). Campylobacteriosis in urban versus rural areas: A case-case study integrated with molecular typing to validate risk factors and to attribute sources of infection. PLoS ONE.

[43] Sibbald C.J., Sharp J.C. (1985). *Campylobacter* infection in urban and rural populations in Scotland. J. Hyg..

[44] Lal A., Ikeda T., French N., Baker M.G., Hales S. (2013). Climate variability, weather and enteric disease incidence in New Zealand: Time series analysis. PLoS ONE.

[45] Oberheim J. (2020). Weather Conditions and Campylobacteriosis in Germany. Ph.D. Dissertation.

[46] Moynihan R., Sanders S., Michaleff Z.A., Scott A.M., Clark J., To E.J., Jones M., Kitchener E., Fox M., Johansson M. (2021). Impact of COVID-19 pandemic on utilisation of healthcare services: A systematic review. BMJ Open.

[47] Karikari A.B., Obiri-Danso K., Frimpong E.H., Krogfelt K.A. (2017). Antibiotic resistance in *Campylobacter* isolated from patients with gastroenteritis in a teaching hospital in Ghana. Open J. Med. Microbiol..

[48] Zachariah O.H., Lizzy M.A., Rose K., Angela M.M. (2021). Multiple drug resistance of *Campylobacter jejuni* and *Shigella* isolated from diarrhoeic children at Kapsabet County referral hospital, Kenya. BMC Infect. Dis..

[49] European Centre for Disease Prevention and Control (ECDC) (2022). Campylobacteriosis. Annual Epidemiological Report for 2021.

[50] Bolinger H., Kathariou S. (2017). The Current State of Macrolide Resistance in *Campylobacter* spp.: Trends and Impacts of Resistance Mechanisms. Appl. Environ. Microbiol..

[51] European Food Safety Authority (EFSA), European Centre for Disease Prevention and Control (ECDC) (2023). The European Union Summary Report on Antimicrobial Resistance in Zoonotic and Indicator Bacteria from Humans, Animals and Food in 2020/2021. EFSA J..

[52] Sithole V., Amoako D.G., Abia A.L.K., Perrett K., Bester L.A., Essack S.Y. (2021). Characterization of *Campylobacter* spp. in Intensive Pig Production in South Africa. Pathogens.

[53] Whitehouse C.A., Zhao S., Tate H. (2018). Antimicrobial resistance in *Campylobacter* species: Mechanisms and genomic epidemiology. Advances in Applied Microbiology.

[54] Nigusu Y., Abdissa A., Tesfaw G. (2022). *Campylobacter* gastroenteritis among under-five children in Southwest Ethiopia. Infect. Drug Resist..

[55] Ansarifar E., Riahi S.M., Tasara T., Sadighara P., Zeinali T. (2023). *Campylobacter* prevalence from food, animals, human and environmental samples in Iran: A systematic review and meta—Analysis. BMC Microbiol..

[56] Dar B. (2010). Prevalence and antimicrobial resistance of *Campylobacter* isolates from humans. Foodborne Pathog. Dis..

[57] Abay K.A., Desalegn G., Weldu Y., Gebrehiwot G.T., Gebreyohannes G., Welekidan L.N., Desta K.H., Asfaw Y.T., Teka A.G., Gebremedhin M.T. (2024). Prevalence and Antimicrobial Resistance of *Campylobacter* Species and Associated Factors Among Under-Five Children with Diarrhea at Randomly Selected Public Health Facilities in Mekelle, Tigray, Ethiopia. Infect. Drug Resist..

[58] Sharifi S., Bakhshi B., Najar-peerayeh S. (2021). Significant contribution of the CmeABC Efflux pump in high-level resistance to ciprofloxacin and tetracycline in *Campylobacter jejuni* and *Campylobacter* coli clinical isolates. Ann. Clin. Microbiol. Antimicrob..

[59] Szczepanska B., Andrzejewska M., Spica D., Klawe J.J. (2017). Prevalence and antimicrobial resistance of *Campylobacter jejuni* and *Campylobacter* coli isolated from children and environmental sources in urban and suburban areas. BMC Microbiol..

[60] Tafa B., Sewunet T., Tassew H., Asrat D. (2014). Isolation and Antimicrobial Susceptibility Patterns of *Campylobacter* Species among Diarrheic Children at Jimma, Ethiopia. Int. J. Bacteriol..

[61] Lengerh A., Moges F., Unakal C., Anagaw B. (2013). Prevalence, associated risk factors and antimicrobial susceptibility pattern of *Campylobacter* species among under five diarrheic children at Gondar University Hospital, Northwest Ethiopia. BMC Pediatr..

[62] Behailu Y., Hussen S., Alemayehu T., Mengistu M., Fenta D.A., Algammal A.M. (2022). Susceptibility patterns of *Campylobacter* infection among under-five children with diarrhea at Governmental Hospitals in Hawassa city, Sidama, Ethiopia. A cross-sectional study. PLoS ONE.

[63] Se M., Joloba M., Kakooza A., Mulindwa K. (2009). *Campylobacter* spp. among Children with acute diarrhea attending Mulago hospital in Kampala-Uganda. Afr. Health Sci..

[64] Autoritatea Europeană pentru Siguranța Alimentară Vizualizarea Datelor Privind Rezistența la Antimicrobiene la Campylobacter. https://multimedia.efsa.europa.eu/dataviz-2022/index.htm.

[65] Abulreesh H.H., Paget T.A., Goulder R. (2006). *Campylobacter* in waterfowl and aquatic environments: Incidence and methods for detection. Environ. Sci. Technol..

[66] Pollett S., Rocha C., Zerpa R., Patiño L., Valencia A., Camiña M., Guevara J., Lopez M., Chuquiray N., Salazar-Lindo E. (2012). *Campylobacter* antimicrobial resistance in Peru: A ten-year observational study. BMC Infect. Dis..

[67] Shin E., Oh Y., Kim M., Jung J., Lee Y. (2013). Antimicrobial resistance patterns and corresponding multilocus sequence types of the *Campylobacter jejuni* isolates from human diarrheal samples. Microb. Drug Resist..

[68] Stockdale A.J., Beeching N.J., Anson J., Beadsworth M.B. (2016). Emergence of extensive fluoroquinolone resistance in *Campylobacter* gastroenteritis in Liverpool, UK. J. Infect..

[69] Schiaffino F., Colston J.M., Paredes-Olortegui M., François R., Pisanic N., Burga R., Peñataro-Yori P., Kosek M.N. (2019). Antibiotic Resistance of *Campylobacter* Species in a Pediatric Cohort Study. Antimicrob. Agents Chemother..

[70] Pham N.T., Thongprachum A., Tran D.N., Nishimura S., Shimizu-Onda Y., Trinh Q.D., Khamrin P., Ukarapol N., Kongsricharoern T., Komine-Aizawa S. (2016). Antibiotic resistance of *Campylobacter jejuni* and *C. coli* isolated from children with diarrhea in Thailand and Japan. Jpn. J. Infect. Dis..

[71] Pan H., Ge Y., Xu H., Zhang J., Kuang D., Yang X., Su X., Huang Z., Shi X., Xu X. (2016). Molecular characterization, antimicrobial resistance and Caco-2 cell invasion potential of *Campylobacter jejuni*/*coli* from young children with diarrhea. Pediatr. Infect. Dis. J..

[72] Ghosh R., Uppal B., Aggarwal P., Chakravarti A., Jha A.K. Increasing antimicrobial resistance of *Campylobacter jejuni* isolated from paediatric diarrhea cases in a tertiary care hospital of New Delhi, India. J. Clin. Diagn. Res..

[73] Cambrea S.C. (2014). Antibiotic susceptibility of Escherichia coli strains isolated in a pediatric population from South Eastern Romania. (Sensibilitatea la antibiotice a tulpinilor de *E. coli* izolate din populaţia pediatrică din sud-estul României). J. Pediatr. Infect. Dis..

[74] Wieczorek K., Osek J. (2015). A five-year study on prevalence and antimicrobial resistance of *Campylobacter* from poultry carcasses in Poland. Food Microbiol..

[75] Taylor N.M., Wales A.D., Ridley A.M., Davies R.H. (2016). Farm level risk factors for fluoroquinolone resistance in *E. coli* and thermophilic *Campylobacter* spp. on poultry farms. Avian Pathol..

[76] Skarp C.P., Hanninen M.L., Rautelin H.I. (2016). Campylobacteriosis: The role of poultry meat. Clin. Microbiol. Infect..

